# Intradiscal quantitative chemical exchange saturation transfer MRI signal correlates with discogenic pain in human patients

**DOI:** 10.1038/s41598-021-97672-y

**Published:** 2021-09-28

**Authors:** Gadi Pelled, Margaux M. Salas, Pei Han, Howard E. Gill, Karl A. Lautenschlager, Tristan T. Lai, Cameron M. Shawver, Matthew B. Hoch, Brandon J. Goff, Aaron M. Betts, Zhengwei Zhou, Cody Lynch, Grant Schroeder, Maxim Bez, Marcel M. Maya, Catherine Bresee, Zulma Gazit, John P. McCallin, Dan Gazit, Debiao Li

**Affiliations:** 1 Board of Governors Regenerative Medicine Institute, Cedars-Sinai Medical Center, Los Angeles, CA 90048 USA; 2 Department of Orthopedics, Cedars-Sinai Medical Center, Los Angeles, CA 90048 USA; 3 Department of Surgery, Cedars-Sinai Medical Center, Los Angeles, CA 90048 USA; 4 Division of Pain Management, Department of Rehabilitation Medicine, Brooke Army Medical Center, San Antonio, TX 78234 USA; 5 59th Medical Wing Air Force, San Antonio, TX 78236 USA; 6 Department of Biomedical Sciences, Cedars-Sinai Medical Center, Los Angeles, CA 90048 USA; 7 Biomedical Research Imaging Institute, Cedars-Sinai Medical Center, Los Angeles, CA 90048 USA; 8 Department of Physical Medicine and Rehabilitation, Uniformed Services University of the Health Sciences, Bethesda, MD 20814 USA; 9 David Geffen School of Medicine, University of California Los Angeles, Los Angeles, CA 90095 USA; 10 Medical Corps, Israel Defense Forces, Tel HaShomer, Israel; 11 Department of Imaging, Cedars-Sinai Medical Center, Los Angeles, CA 90048 USA; 12 Biostatistics and Bioinformatics Research Center, Samuel Oschin Comprehensive Cancer Institute, Cedars-Sinai Medical Center, Los Angeles, CA 90048 USA; 13 Faculty of Dental Medicine, Hebrew University, 91120 Jerusalem, Israel; 14 Department of Bioengineering, University of California Los Angeles, Los Angeles, CA 90095 USA

**Keywords:** Biomarkers, Translational research, Magnetic resonance imaging

## Abstract

Low back pain (LBP) is often a result of a degenerative process in the intervertebral disc. The precise origin of discogenic pain is diagnosed by the invasive procedure of provocative discography (PD). Previously, we developed quantitative chemical exchange saturation transfer (qCEST) magnetic resonance imaging (MRI) to detect pH as a biomarker for discogenic pain. Based on these findings we initiated a clinical study with the goal to evaluate the correlation between qCEST values and PD results in LBP patients. Twenty five volunteers with chronic low back pain were subjected to T2-weighted (T2w) and qCEST MRI scans followed by PD. A total of 72 discs were analyzed. The average qCEST signal value of painful discs was significantly higher than non-painful discs (*p* = 0.012). The ratio between qCEST and normalized T2w was found to be significantly higher in painful discs compared to non-painful discs (*p* = 0.0022). A receiver operating characteristics (ROC) analysis indicated that qCEST/T2w ratio could be used to differentiate between painful and non-painful discs with 78% sensitivity and 81% specificity. The results of the study suggest that qCEST could be used for the diagnosis of discogenic pain, in conjunction with the commonly used T2w scan.

## Introduction

Low back pain (LBP) is one of the most common causes of surgical procedures and one of the most frequent reasons for doctors’ visits and hospital admissions^1,2^. For the general US adult population, the reported lifetime prevalence of LBP ranges from 65 to 80%^3^. Most often, LBP is a result of the degenerative process of the intervertebral disc (IVD) that occurs as a natural part of aging, but LBP can also occur as a result of congenital disorders, mechanical injuries, and certain lifestyle factors^4,5^. In over 90% of surgical spine procedures, however, the spontaneous degeneration of the IVD is believed to be the source of chronic LBP^6^.

Identification of the precise component of a patient’s LBP—whether discogenic, radicular, muscular, vertebral body, facet joint, sacroiliac joint, or spinal stenosis—is highly desired in order to offer appropriate treatment interventions. However, evidence suggests that clinicians have a low accuracy of subclassifying LBP, which likely leads to unsuccessful treatment attempts^7^. Invasive methods of diagnosing discogenic LBP include anesthetic discography or “discoblock” and provocative discography (PD); which is the current gold standard. PD involves X-ray findings of disc defects, the injection of contrast agent into the IVD under X-ray guidance, and a positive elicitation of pain, suggesting a diagnosis of LBP originating from disc damage or degeneration. Pain associated with this procedure can last for over a year in some patients^8^, which prompted the development of anesthetic discography, whereby a local anesthetic is injected into the disc, and relief of pain is considered a positive result for discogenic back pain. Several studies have suggested that anesthetic discography may have the potential to replace the diagnostic role of PD^9,10^, however current guidelines by the North American Spine Society conclude there is insufficient evidence to make a recommendation for or against anesthetic discography^11^. These same guidelines suggest that PD currently remains the most accurate diagnostic modality for identifying discogenic pain, citing high-level evidence that PD correlates with other robust diagnostic modalities such as pain reproduction in the presence of disc degeneration on MRI/CT discography and the presence of vertebral endplate abnormalities on MRI imaging^11^. Despite remaining the gold standard diagnostic test for discogenic LBP, the popularity of PD has declined in recent years, likely due to the invasiveness of the procedure, concerns about the potential to cause IVD injury^12^, and unclear value in predicting surgery outcomes^13,14^.

Noninvasive methods for the diagnosis of discogenic LBP, which generally include different magnetic resonance imaging modalities, have the advantage of avoiding disc injection and the associated risk of disc damage, pain, and infection. Conventional MRI morphological features including Pfirrmann grading of IVD degeneration and findings such as a high-intensity zone on T2 weighted MRI in the anterior or posterior annulus fibrosus have been suggested to have associations with discogenic LBP^15–19^, however other reports have shown no such correlation and a high prevalence of these changes in asymptomatic patients^14,20–23^. Newer magnetic resonance methods such as magnetic resonance spectroscopy (MRS) have been developed to noninvasively evaluate biochemical disc composition. These methods were created in response to the emerging hypothesis that acidification of IVDs is the primary driver of discogenic LBP. A recent clinical study of MRS to identify painful discs showed a strong concordance with PD, and a 97% successful surgical outcome for positive discs versus 57% for negative discs^24^. However, in vivo MRS suffers from limited signal-to-noise ratio (SNR), physiological motion, and bone susceptibility induced line broadening^25^.

Chemical exchange saturation transfer (CEST) exploits the pH sensitive chemical change, which occurs between water protons and solute protons and has previously been studied in IVDs in pigs and humans. Unfortunately, CEST has confounding influence by water relaxation parameters and solute concentration, which has lead to the evolvement of quantitative CEST (qCEST)^26–28^. qCEST is capable of measuring exchange rate, independent of T_1_, T_2_, and solute concentration by measuring the pH-sensitive exchange rate of glycosaminoglycans’ (GAGs) hydroxyl protons in the nucleus pulposus (NP) and water protons^29–31^. We previously showed that the use of qCEST in a pig model of IVD degeneration resulted in a strong positive correlation between the expression of pain markers and increase in qCEST signal, which was correlated with a significant decrease of pH inside degenerated IVDs^29,32^. Based on our results in the porcine disc degeneration model, we initiated a clinical study with the goal to evaluate the correlation between qCEST values and discography results in LBP patients.

## Methods

### Patient enrolment

The Institutional Review Boards approved all experiments and each subject provided informed consent (IRB 59th Medical Wing #: FWH20170104H; Cedars-Sinai Medical Center #37795). All research was performed in accordance with relevant guidelines and regulations approved by both Review Boards.

**Figure 1:**
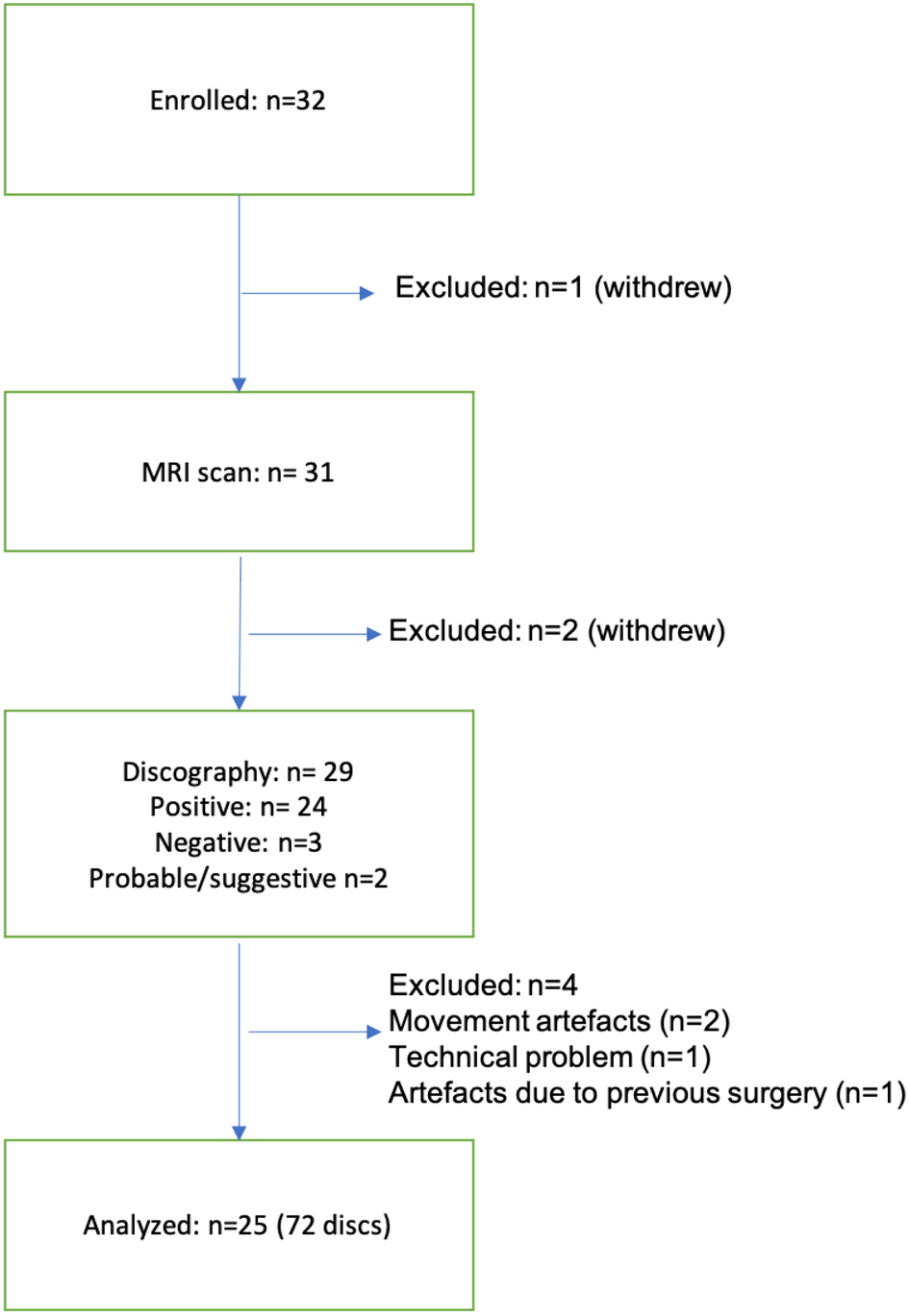
Flowchart of participants.

A total of 32 volunteers who suffered from chronic LBP volunteered to take part in the study. The complete inclusion and exclusion criteria are detailed in Table 1. The demographics of the volunteers are detailed in Table 2. Only patients with back pain due to degenerative disc disease without significant disc herniation or radicular leg pain were included. Three volunteers withdrew from the study prior to MRI or PD. An additional four volunteers were excluded from the final analysis due to artifacts during MRI acquisition that were attributed to patient movement and prior back surgery. Each patient underwent an MRI in which qCEST and T2-weighted (T2w) measurements were performed. Between two and four weeks post-MRI, each patient underwent a multi-level provocative discography. The results of the discography were compared to the values obtained from the MRI scan from 25 subjects (Fig. 1).

**Table 1:** Inclusion and exclusion criteria.

Inclusion criteria	• Subjects who are at least 21 years of age and no older than 70 years of age; of either gender and in good general health • Subjects with chronic lumbar back pain for 6 months or greater duration due to moderate degenerative disc disease in any lumbar vertebral level between L1 and S1 • Subjects must have failed at least 3 months of non-operative management for low back pain with exposure to physical therapy • Low back pain must be at least 40 mm out of a 100 mm on the Visual Analog Scale with either leg having pain less than back pain and non-radicular origin • Lumbar disc pathology must have a modified Pfirrmann score of 3, 4, 5, or 6 with a herniation no greater than 6 mm and no neurological compression • Pain/pathology must not originate from facet joints or stenosis
Exclusion criteria	• Subjects with ferromagnetic materials within the body • Pregnant or lactating females • Inability to undergo an MRI • Excessive abdominal girth preventing entrance into the magnet bore

**Table 2:** Demographics of patients included in the study.

Characteristic	*N* = 25
Male, *N* (%)	20 (80)
Age, mean (range)	35.8 (25–42)
Pain duration > 1 year, *N* (%)	24 (96)
Race, *N* (%)	
White	19 (76)
Black	4 (16)
Asian	1 (4)
Not specified	1 (4)
Ethnicity, *N* (%)	
Non-Hispanic	12 (48)
Hispanic	5 (20)
Not specified	8 (32)
Highest educational grade, mean ± SD	14.3 ± 1.7

### Pain evaluation

Defense and Veterans Pain Rating Scale (DVPRS) form and a numeric pain questionnaires were completed by participants before and after MRI and PD. The DVPRS provides a qualitative score on pain during everyday living: activity, sleep, mood, and stress for 24-h on a scale of 1 to 10 (0–4 = mild pain, 5–6 = moderate, 7–10 = severe pain). The numeric pain questionnaire was given to assess current pain, worst pain in the last 7 days, average pain in the last 7 days, and the interference of pain on activity, sleep, mood, and stress in the last 24 h.

### MRI protocol

MRI was performed using a 3 T clinical MRI system (Magnetom Verio; Siemens Healthcare, Erlangen, Germany). qCEST MRI and T2w imaging (total scan time—45 min) were performed in the sagittal plane to cover the discs of interest. For the qCEST imaging, images were acquired using a two-dimensional reduced-field-of-view single-shot TSE CEST sequence with TR/TE = 5000/8.7 ms, 5 averages, FOV = 220 × 69 mm^2^, and spatial resolution = 1.7 × 1.7 × 8.0 mm^3^^33^. The CEST saturation module consisted of 39 Gaussian-shaped pulses, with a duration t_p_ = 80 ms for each pulse and an interpulse delay t_d_ = 80 ms (duty cycle = 50%, total saturation duration T_s_ = 6240 ms) at saturation flip angles of 900, 1500, 2100, and 3000 [B_1_ amplitudes = flip angle/(gt_p_) = 0.73, 1.22, 1.71, and 2.45 µT]; the Z-spectrum was acquired with 13 different saturation frequencies from − 1.8 to 1.8 ppm as well as unsaturated reference S_0_. T2w images were acquired using a TSE sequence with TR/TE = 3620/100 ms, FOV = 270 × 270 mm^2^, and spatial resolution = 0.84 × 0.84 × 4 mm^3^.

### Discography procedure

Provocation discography followed the guidelines as set by the Spine Interventions Society in the 2013 Practice Guidelines for Spinal Diagnostic and Treatment Procedures. Periprocedure antibiotics were given to all patients either intravenously and/or intradiscally utilizing cefazolin sodium or clindamycin. The selected intervertebral disc levels were typically the last three lower lumbar discs as the L4-5 and/or L5-S1 segments are more frequently symptomatic while the L3-4 disc is frequently asymptomatic. Providers reviewed the standard lumbosacral spine MRI before provocation discography was pursued, and choice of disc levels and/or interrogation of the discs occurred. Patients were then prepped and draped in sterile fashion, and after appropriate skin and subcutaneous anesthetic, a 22-gauge needle was inserted under fluoroscopic guidance into the center of the three lower lumbar discs using a posterolateral approach. In preparation for discs stimulation, a needle was placed into the central region of the 3 lower discs. Interrogation of these discs occurred in the order of least likely, to most likely to provoke concordant discogenic pain. Generally, the L3-4 disc would be assessed initially and the lower two discs would be evaluated subsequently. Utilizing a manometer, contrast medium was injected slowly and intradiscal pressures noted and recorded for both the opening pressure and pressure at pain provocation. Injection would continue until concordant pain was reproduced, contrast medium escaped from the disc, a volume of 3 mL’s was reached, or a maximum pressure of 50 psi above opening pressure was obtained. At least one nonpainful level was pursued to confirm the internal validity of the tests. A post procedure lumbar CT scan was then acquired for all patients. Subsequently, the data for each disc stimulation was collected to determine the likelihood of discogenic pain from that level utilizing the Spine Interventional Society criteria. The data included the disc level, the opening pressure, the pressure for pain reproduction or discontinuation, the pre-and post-provocation, DVPRS, numeric pain levels, and the modified Dallas discography criteria.

### Data analysis

Postprocessing was performed with custom-written programs in MATLAB (MathWorks, Natick, MA, USA). Regions of interest (ROIs) were placed to encompass the central area of nucleus pulposus at each disc. Manual ROI selection was performed for each disc of the spine within CEST and T2w images.

For qCEST, the Z-spectra acquired at each saturation power were first fitted with three-pool (water, MT, gagCEST) Lorentzian model to correct B_0_ inhomogeneities and to extract the gagCEST signal for the $\Omega$-plot analysis^34^. Then the qCEST signal (the exchange rate ${k}_{sw}$, in s^−1^) was estimated for each disc, as was previously described^32^. Rather than pixel-wise analysis in Zhou et al.^29^, the qCEST fitting was done using the average signal within each ROI to improve the robustness.

For T2w images, the T2w signal intensities were averaged within each ROI, and then normalized with the average signal intensity within the vertebra region. Averaged and normalized T2w image intensities of each ROI were used for further analysis.

### Statistical analysis

The ratio between qCEST and T2w was calculated per each disc. Average ratio scores were tested across painful and non-painful rated spinal discs with mixed model regression to account for the repeated measures within patients. Generalized linear mixed model regression was used for repeated measures logistic regression using empirical estimation and an unstructured covariance. Receiver operation characteristic (ROC) curves were constructed with area under the curve (AUC) estimated. Optimal qCEST/T2w cutpoint was established based on maximal Youden Index value. Statistical analysis was performed using SPSS v. 16.0 (SPSS, Chicago, IL) and SAS v. 9.4 (SAS, Cary, NC, USA).

## Results

### Pain scores

**Figure 2:**
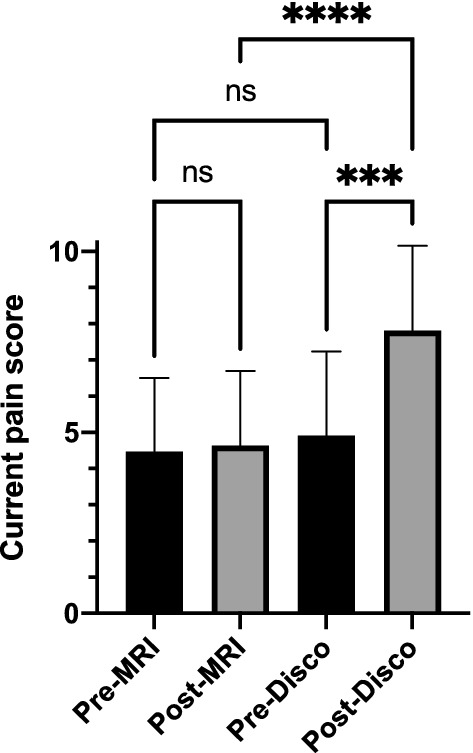
Pain scores. Pain scores were assessed by questionnaires before and after MRI and PD. Mean pain scores for Pre-MRI, Post-MRI, Pre-Discogram (Pre-Disco), and Post-Discogram (Post-Disco) were 4.5, 4.6, 4.9, and 7.8 respectively. Scores were significantly higher post PD compared to pre-PD and post-MRI scans (***p = 0.0001; ****p < 0.0001, n = 25).

Analysis of pain scores based on DVPRS questionnaires and numeric pain scales showed that pain levels were similar prior to and post MRI scans. However, scores increased significantly post PD compared to pre-PD and to post MRI scan (Fig. 2).

### Correlation between MRI signal and PD results

**Figure 3:**
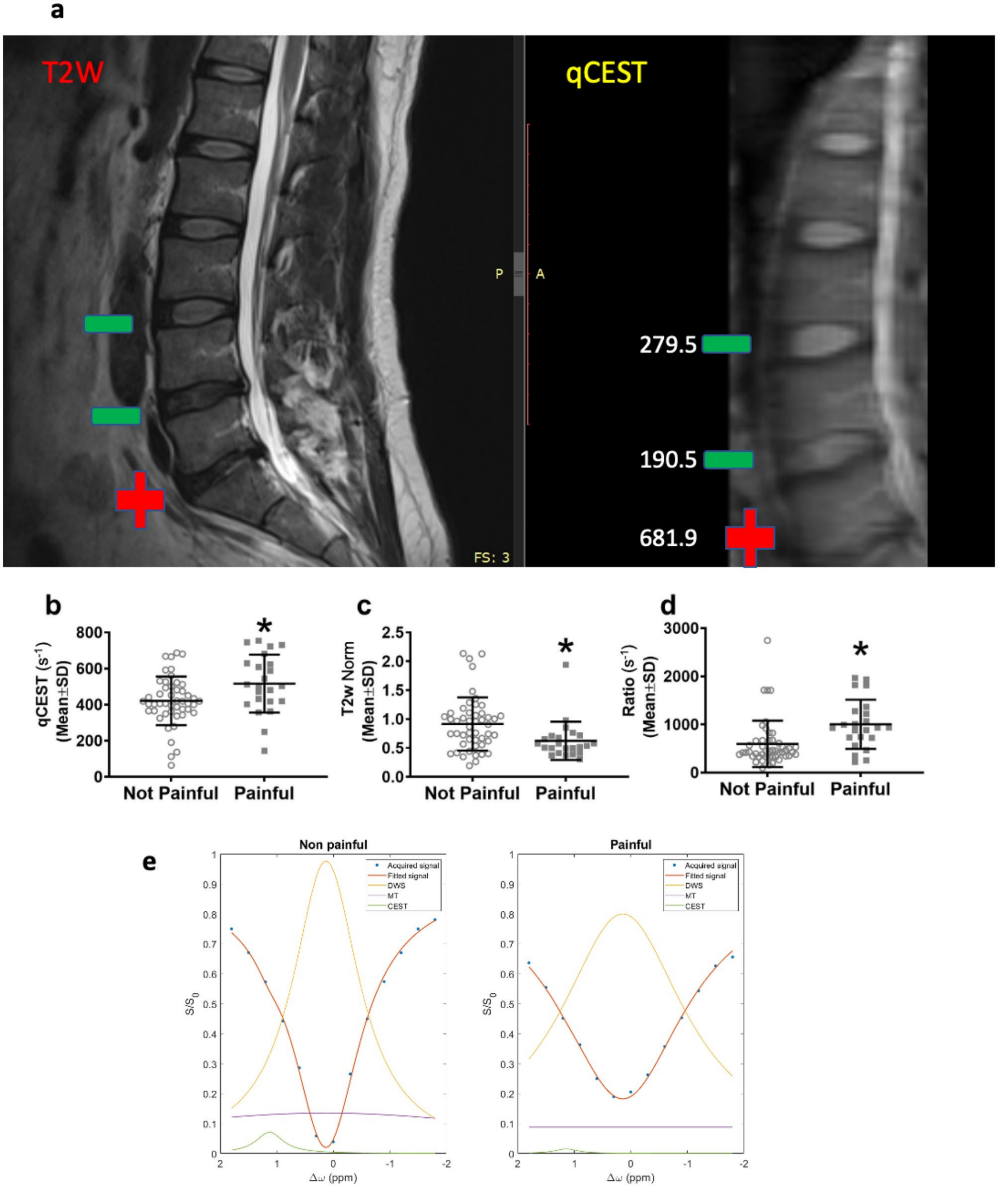
QCEST, T2W and qCEST/T2W ratio values were significantly different between painful and non-painful discs. Representative T2W and qCEST MRI scans of a human patient showing a disc that was determined painful in PD (+) and discs that were non-painful in PD (−). qCEST values are indicated for each disc (**a**). The average qCEST value of painful discs was significantly higher than non-painful discs (*p* = 0.012; **b**). The average normalized T2w value of painful discs was significantly lower than non-painful discs (*p* = 0.0091; **c**). The ratio between qCEST and normalized T2w was calculated for each disc and was found to be significantly higher in painful discs compared to non-painful discs (*p* = 0.0022; **d**). (n = 72 discs). Representative Z-spectra (acquired at saturation flip angles of 900 or effective B_1_ = 0.73 µT) of painful (L5/S1) and non-painful (L3/L4) discs (**e**) (Norm = normalized).

A total of 72 discs from 25 patients were acquired and assessed for pain using discography (median = 3/patient, range: 1–4). 32% of the discs evaluated (23/72) were scored by discography as painful (Fig. 3a). The average qCEST value of painful discs was significantly higher than non-painful discs (516.6 ± 160.3 and 421.2 ± 134.5, respectively; *p* = 0.012; Fig. 3b). The average normalized T2w value of painful discs was significantly lower than non-painful discs (0.62 ± 0.33 and 0.92 ± 0.46, respectively; *p* = 0.0091; Fig. 3c). The ratio between qCEST and normalized T2w was calculated for each disc and was found to be significantly higher in painful discs compared to non-painful discs (1003 ± 509.9) and 596 ± 483.3, respectively; *p* = 0.0022; Fig. 3d). Representative Z-spectra generated from painful (L5/S1) and non-painful (L3/L4) discs show lower gagCEST signal in the painful disc (Fig. 3e).

**Figure 4:**
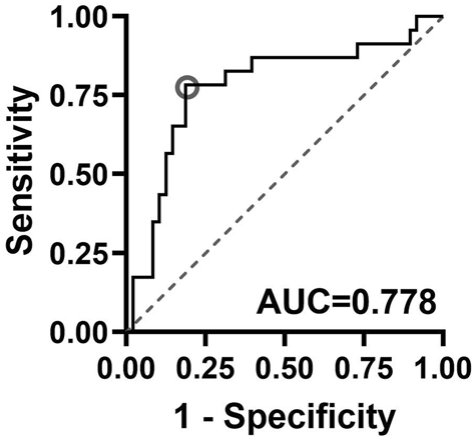
Sensitivity and specificity of qCEST/T2w ratio to differentiate painful from non-painful discs. A receiver operating characteristics (ROC) analysis indicated that qCEST/normalized T2w ratio could be used to differentiate between painful and non-painful discs at a cutpoint of > 642 with 78% sensitivity and 81% specificity [AUC = 0.778 (95% CI 0.6511, 0.9051)].

In order to assess the utility of the MRI biomarker in detecting low back pain, a receiver operating characteristics (ROC) analysis was performed which indicated that qCEST/normalized T2w ratio could be used to differentiate between painful and non-painful discs at a cutpoint of > 642 with 78% sensitivity and 81% specificity [AUC = 0.778 (95% CI: 0.6511, 0.9051)); Fig. 4]. Sensitivity and specificity was 83% and 69% respectively with a ratio cutpoint of > 514.

## Discussion

We sought to examine if qCEST MRI could accurately detect painful discs in LBP patients that were candidates for PD. Our results showed that painful IVDs had significantly higher qCEST scores than non-painful IVDs. Interestingly, when the ratio between the qCEST signal to the normalized T2w signal was calculated, it yielded an even more significant difference between painful to non-painful discs. ROC curve for qCEST/T2 ratio showed 78% sensitivity and 81% specificity to detect painful discs. The high sensitivity of qCEST/T2w ratio makes it particularly useful for ruling out patients with a low qCEST/T2w score for discogenic LBP who would otherwise will be referred to PD. For example, at the cutpoint of qCEST/normalized T2w ratio of > 642 for predicting PD positive reaction, 39 of the 72 discs considered in this study with a score ≤ 642 could have avoided having to undergo PD, with only 5 false negatives and a negative predictive value (NPV) of 88%. Changing the cutpoint to > 514 could increase the sensitivity to 83% and NPV to 89%, while decreasing the specificity to 69%. It is worth mentioning that an artificially high prevalence of discogenic LBP in many studies of this subject often inflates positive predictive values, which would otherwise be lower in the setting of a realistic patient population with a lower prevalence. On the other hand, negative predictive values increase as prevalence decreases, pointing to the likely higher performance of the qCEST/T2w for predicting PD—in an even wider population of LBP patients than those included in this study.

A potential limitation of this study is the relatively small sample size of 25 patients and 72 discs. Furthermore, we considered PD—discs from LBP patients as normal/non-painful rather than including asymptomatic patient discs as controls. Additionally, the patients in this study had an average age slightly lower than other studies and were also predominantly male. Finally, we used PD as a reference standard for diagnosing discogenic LBP, when its accuracy has been questioned with reports of sensitivity and specificity ranging from 25–100% and 64–100% respectively when compared to other diagnostic methods including spine surgery success^35,36^. That being said, we felt that success of spine surgery introduced more confounding variables than it eliminated, and thus decided to compare the performance of qCEST MRI to PD, the current gold standard of diagnosing discogenic LBP.

As more disc-targeted therapies are being developed and applied to treat discogenic pain, so grows the need for an objective imaging method to monitor pain elimination. Examples for these methods include ablation of the basivertebral nerve^37,38^ and stem cell injections. Orozco et al. injected a single dose of autologous bone marrow-derived MSCs to patients and showed an improvement in their pain and disability scores^39^. Other works using hematopoietic stem cells^40^ and allogeneic juvenile chondrocytes^41^ reported an improvement in pain scores. Currently, an ongoing Phase III clinical trial is evaluating the effect of mesenchymal precursor cells on 404 subjects with chronic LBP (ClinicalTrials.gov Identifier: NCT02412735). However, these studies used patient-dependent self-assessment questionnaires, which are subjective and prone to bias^42^. The qCEST method could serve as a reliable quantitative method to monitor the progression of pain relief. We have conducted a small pilot study in minipigs with degenerate discs and showed that a single injection of induced pluripotent stem cell-derived notochordal cells reduced the signal of qCEST over time^43^. Although no pain markers were assessed in that pilot study, we have previously shown that such a model of disc degeneration was associated with an up-regulation in inflammatory and pain markers^32^.

Finally, it is foreseeable that in the future a single MRI protocol will provide the physician with variable information on the patient’s IVD status. Specifically, it should be a quantitative imaging tool that will be able to generate data on pain, extra cellular matrix, hydration, disc height, and anatomical changes. One potential tool could be the recently developed Multitasking MRI technique^44–48^, which allows efficient and simultaneous multiparametric mapping by taking advantage of the vast data redundancy between various parameters. So far it has been applied to motion-resolved, free breathing T1 and T1 mapping of the heart^44,45^, quantitative contrast‐enhanced MR imaging of carotid vessel wall^46^ and pancreas^48^ using dynamic T1 mapping, and simultaneous T1, T2, and ADC mapping in the brain^47^. Multitasking MRI, for the IVD could potentially include qCEST, T1, T2, ADC, and more.

In conclusion, our results show that the average qCEST value of painful discs was significantly higher than non-painful discs. Additionally, we found the average normalized T2w value of painful discs was significantly lower than non-painful discs. We determined the ratio of qCEST value to normalized T2w value as the most useful quantitative measurement in predicting painful versus non-painful discs, with a 78% sensitivity and 81% specificity, or an 83% sensitivity and 69% specificity, depending on the selected cutpoint. We believe the clinical utility of the qCEST/normalized T2w ratio is maximized around a sensitivity of 83%, which was associated with a NPV of 89% in our patient sample. This high sensitivity—in contrast to a variety of MRI findings associated with discogenic back pain such as disc protrusion, endplate abnormalities, or high intensity zone (HIZ) that are specific but poorly sensitive^49–53^—makes the qCEST/normalized T2w ratio useful for ruling out discogenic LBP in patients with low scores, and sparing them from the painful and potentially damaging procedure of PD.
